# Approaching literature review for academic purposes: The Literature Review Checklist

**DOI:** 10.6061/clinics/2019/e1403

**Published:** 2019-11-19

**Authors:** Debora F.B. Leite, Maria Auxiliadora Soares Padilha, Jose G. Cecatti

**Affiliations:** I Departamento de Ginecologia e Obstetricia, Faculdade de Ciencias Medicas, Universidade Estadual de Campinas, Campinas, SP, BR; II Universidade Federal de Pernambuco, Pernambuco, PE, BR; III Hospital das Clinicas, Universidade Federal de Pernambuco, Pernambuco, PE, BR

**Keywords:** Review, Checklist, Academic Performance, Critical Thinking, Learning

## Abstract

A sophisticated literature review (LR) can result in a robust dissertation/thesis by scrutinizing the main problem examined by the academic study; anticipating research hypotheses, methods and results; and maintaining the interest of the audience in how the dissertation/thesis will provide solutions for the current gaps in a particular field. Unfortunately, little guidance is available on elaborating LRs, and writing an LR chapter is not a linear process. An LR translates students’ abilities in information literacy, the language domain, and critical writing. Students in postgraduate programs should be systematically trained in these skills. Therefore, this paper discusses the purposes of LRs in dissertations and theses. Second, the paper considers five steps for developing a review: defining the main topic, searching the literature, analyzing the results, writing the review and reflecting on the writing. Ultimately, this study proposes a twelve-item LR checklist. By clearly stating the desired achievements, this checklist allows Masters and Ph.D. students to continuously assess their own progress in elaborating an LR. Institutions aiming to strengthen students’ necessary skills in critical academic writing should also use this tool.

## INTRODUCTION

Writing the literature review (LR) is often viewed as a difficult task that can be a point of writer’s block and procrastination (1) in postgraduate life. Disagreements on the definitions or classifications of LRs (2) may confuse students about their purpose and scope, as well as how to perform an LR. Interestingly, at many universities, the LR is still an important element in any academic work, despite the more recent trend of producing scientific articles rather than classical theses.

The LR is not an isolated section of the thesis/dissertation or a copy of the background section of a research proposal. It identifies the state-of-the-art knowledge in a particular field, clarifies information that is already known, elucidates implications of the problem being analyzed, links theory and practice (3-5), highlights gaps in the current literature, and places the dissertation/thesis within the research agenda of that field. Additionally, by writing the LR, postgraduate students will comprehend the structure of the subject and elaborate on their cognitive connections (3) while analyzing and synthesizing data with increasing maturity.

At the same time, the LR transforms the student and hints at the contents of other chapters for the reader. First, the LR explains the research question; second, it supports the hypothesis, objectives, and methods of the research project; and finally, it facilitates a description of the student’s interpretation of the results and his/her conclusions. For scholars, the LR is an introductory chapter (6). If it is well written, it demonstrates the student’s understanding of and maturity in a particular topic. A sound and sophisticated LR can indicate a robust dissertation/thesis.

**Figure 1:**
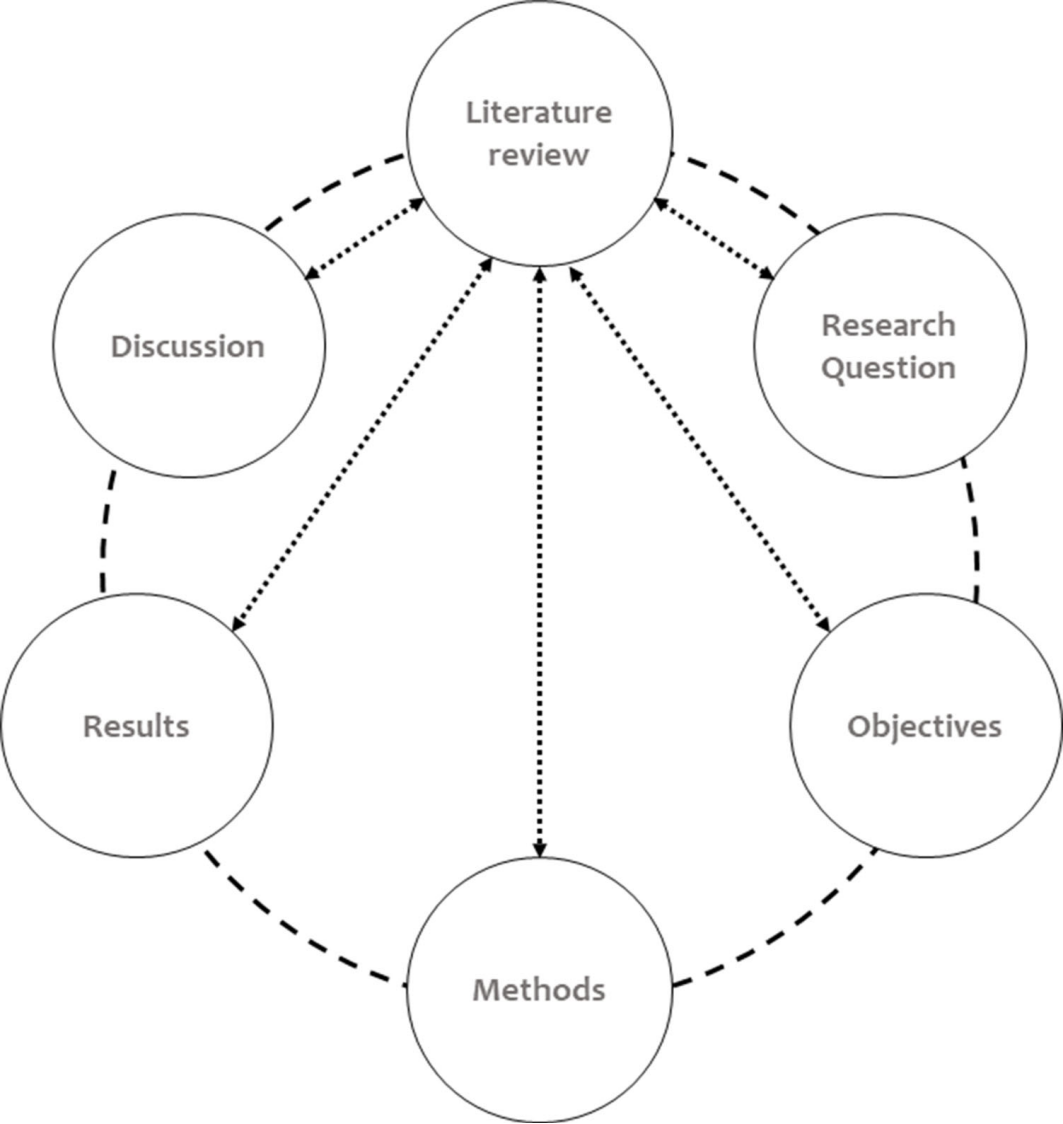
The LR chapter is an elemental component of thesis and dissertations, and it is directly connected to other sections. By assessing the LR chapter, the reader might anticipate what to expect from the remaining sections of the academic text.

A consensus on the best method to elaborate a dissertation/thesis has not been achieved. The LR can be a distinct chapter or included in different sections; it can be part of the introduction chapter, part of each research topic, or part of each published paper (7). However, scholars view the LR as an integral part of the main body of an academic work because it is intrinsically connected to other sections (Figure 1) and is frequently present. The structure of the LR depends on the conventions of a particular discipline, the rules of the department, and the student’s and supervisor’s areas of expertise, needs and interests.

Interestingly, many postgraduate students choose to submit their LR to peer-reviewed journals. As LRs are critical evaluations of current knowledge, they are indeed publishable material, even in the form of narrative or systematic reviews. However, systematic reviews have specific patterns^1^ (8) that may not entirely fit with the questions posed in the dissertation/thesis. Additionally, the scope of a systematic review may be too narrow, and the strict criteria for study inclusion may omit important information from the dissertation/thesis. Therefore, this essay discusses the definition of an LR is and methods to develop an LR in the context of an academic dissertation/thesis. Finally, we suggest a checklist to evaluate an LR.

## WHAT IS A LITERATURE REVIEW IN A THESIS?

Conducting research and writing a dissertation/thesis translates rational thinking and enthusiasm (9). While a strong body of literature that instructs students on research methodology, data analysis and writing scientific papers exists, little guidance on performing LRs is available. The LR is a unique opportunity to assess and contrast various arguments and theories, not just summarize them. The research results should not be discussed within the LR, but the postgraduate student tends to write a comprehensive LR while reflecting on his or her own findings (10).

Many people believe that writing an LR is a lonely and linear process. Supervisors or the institutions assume that the Ph.D. student has mastered the relevant techniques and vocabulary associated with his/her subject and conducts a self-reflection about previously published findings. Indeed, while elaborating the LR, the student should aggregate diverse skills, which mainly rely on his/her own commitment to mastering them. Thus, less supervision should be required (11). However, the parameters described above might not currently be the case for many students (11,12), and the lack of formal and systematic training on writing LRs is an important concern (11).

An institutional environment devoted to active learning will provide students the opportunity to continuously reflect on LRs, which will form a dialogue between the postgraduate student and the current literature in a particular field (13). Postgraduate students will be interpreting studies by other researchers, and, according to Hart (1998) (3), the outcomes of the LR in a dissertation/thesis include the following:

- To identify what research has been performed and what topics require further investigation in a particular field of knowledge;
- To determine the context of the problem;
- To recognize the main methodologies and techniques that have been used in the past;
- To place the current research project within the historical, methodological and theoretical context of a particular field;
- To identify significant aspects of the topic;
- To elucidate the implications of the topic;
- To offer an alternative perspective;
- To discern how the studied subject is structured;
- To improve the student’s subject vocabulary in a particular field; and
- To characterize the links between theory and practice.

A sound LR translates the postgraduate student’s expertise in academic and scientific writing: it expresses his/her level of comfort with synthesizing ideas (11). The LR reveals how well the postgraduate student has proceeded in three domains: an effective literature search, the language domain, and critical writing.

### Effective literature search

All students should be trained in gathering appropriate data for specific purposes, and information literacy skills are a cornerstone. These skills are defined as “an individual’s ability to know when they need information, to identify information that can help them address the issue or problem at hand, and to locate, evaluate, and use that information effectively” (14). Librarian support is of vital importance in coaching the appropriate use of Boolean logic (AND, OR, NOT) and other tools for highly efficient literature searches (e.g., quotation marks and truncation), as is the appropriate management of electronic databases.

### Language domain

Academic writing must be concise and precise: unnecessary words distract the reader from the essential content (15). In this context, reading about issues distant from the research topic (16) may increase students’ general vocabulary and familiarity with grammar. Ultimately, reading diverse materials facilitates and encourages the writing process itself.

### Critical writing

Critical judgment includes critical reading, thinking and writing. It supposes a student’s analytical reflection about what he/she has read. The student should delineate the basic elements of the topic, characterize the most relevant claims, identify relationships, and finally contrast those relationships (17). Each scientific document highlights the perspective of the author, and students will become more confident in judging the supporting evidence and underlying premises of a study and constructing their own counterargument as they read more articles. A paucity of integration or contradictory perspectives indicates lower levels of cognitive complexity (12).

Thus, while elaborating an LR, the postgraduate student should achieve the highest category of Bloom’s cognitive skills: evaluation (12). The writer should not only summarize data and understand each topic but also be able to make judgments based on objective criteria, compare resources and findings, identify discrepancies due to methodology, and construct his/her own argument (12). As a result, the student will be sufficiently confident to show his/her own *voice*.

Writing a consistent LR is an intense and complex activity that reveals the training and long-lasting academic skills of a writer. It is not a lonely or linear process. However, students are unlikely to be prepared to write an LR if they have not mastered the aforementioned domains (10). An institutional environment that supports student learning is crucial.

Different institutions employ distinct methods to promote students’ learning processes. First, many universities propose modules to develop *behind the scenes* activities that enhance self-reflection about general skills (e.g., the skills we have mastered and the skills we need to develop further), behaviors that should be incorporated (e.g., self-criticism about one’s own thoughts), and each student’s role in the advancement of his/her field. Lectures or workshops about LRs themselves are useful because they describe the purposes of the LR and how it fits into the whole picture of a student’s work. These activities may explain what type of discussion an LR must involve, the importance of defining the correct scope, the reasons to include a particular resource, and the main role of critical reading.

Some pedagogic services that promote a continuous improvement in study and academic skills are equally important. Examples include workshops about time management, the accomplishment of personal objectives, active learning, and foreign languages for nonnative speakers. Additionally, opportunities to converse with other students promotes an awareness of others’ experiences and difficulties. Ultimately, the supervisor’s role in providing feedback and setting deadlines is crucial in developing students’ abilities and in strengthening students’ writing quality (12).

## HOW SHOULD A LITERATURE REVIEW BE DEVELOPED?

**Figure 2:**
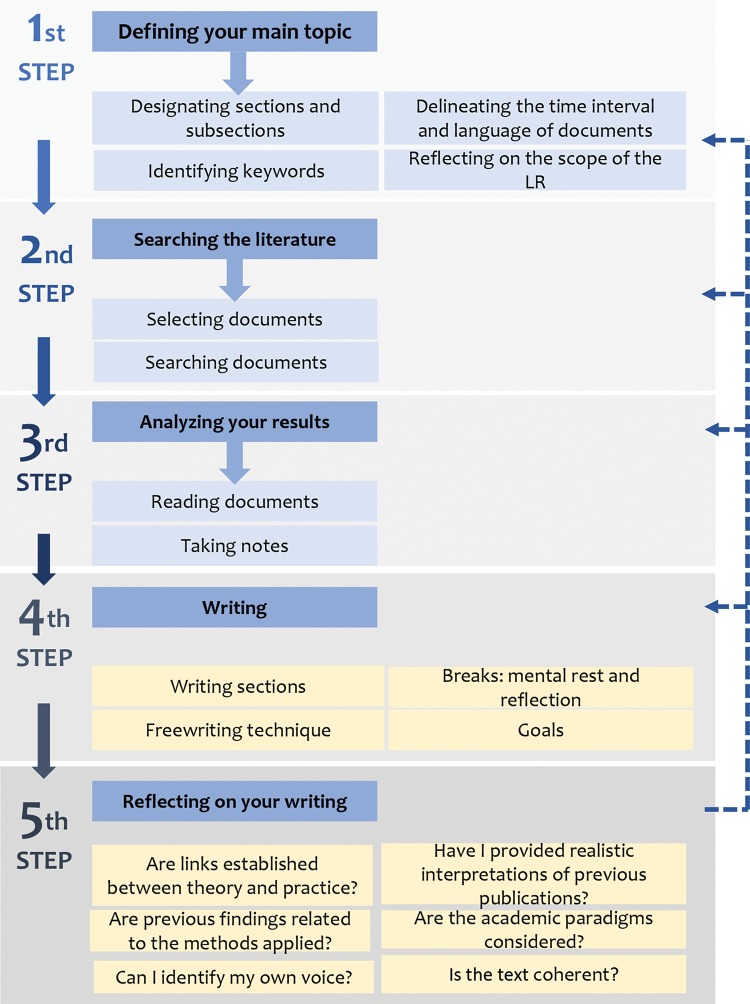
The five steps in performing a solid LR for dissertations or thesis. The first three steps are divided into subsections, the fourth step suggests writing strategies, and the fifth step comprises some signaling questions to practice and evaluate critical writing. These steps are not a straightforward rule, and previous steps may need to be repeated to improve the quality of the LR.

A consensus on the appropriate method for elaborating an LR is not available, but four main steps are generally accepted: defining the main topic, searching the literature, analyzing the results, and writing (6). We suggest a fifth step: reflecting on the information that has been written in previous publications (Figure 2).

### First step: Defining the main topic

Planning an LR is directly linked to the research main question of the thesis and occurs in parallel to students’ training in the three domains discussed above. The planning stage helps organize ideas, delimit the scope of the LR (11), and avoid the wasting of time in the process. Planning includes the following steps:

- Reflecting on the scope of the LR: postgraduate students will have assumptions about what material must be addressed and what information is not essential to an LR (13,18). Cooper’s Taxonomy of Literature Reviews^2^ systematizes the writing process through six characteristics and nonmutually exclusive categories. The *focus* refers to the reviewer’s most important points of interest, while the *goals* concern what students want to achieve with the LR. The *perspective* assumes answers to the student’s own view of the LR and how he/she presents a particular issue. The *coverage* defines how comprehensive the student is in presenting the literature, and the *organization* determines the sequence of arguments. The *audience* is defined as the group for whom the LR is written.
- Designating sections and subsections: Headings and subheadings should be specific, explanatory and have a coherent sequence throughout the text (4). They simulate an inverted pyramid, with an increasing level of reflection and depth of argument.
- Identifying keywords: The relevant keywords for each LR section should be listed to guide the literature search. This list should mirror what Hart (1998) (3) advocates as *subject vocabulary*. The keywords will also be useful when the student is writing the LR since they guide the reader through the text.
- Delineating the time interval and language of documents to be retrieved in the second step. The most recently published documents should be considered, but relevant texts published before a predefined cutoff year can be included if they are classic documents in that field. Extra care should be employed when translating documents.

### Second step: Searching the literature

The ability to gather adequate information from the literature must be addressed in postgraduate programs. Librarian support is important, particularly for accessing difficult texts. This step comprises the following components:

- Searching the literature itself: This process consists of defining which databases (electronic or dissertation/thesis repositories), official documents, and books will be searched and then actively conducting the search. Information literacy skills have a central role in this stage. While searching electronic databases, controlled vocabulary (e.g., Medical Subject Headings, or MeSH, for the PubMed database) or specific standardized syntax rules may need to be applied.

In addition, two other approaches are suggested. First, a review of the reference list of each document might be useful for identifying relevant publications to be included and important opinions to be assessed. This step is also relevant for referencing the original studies and leading authors in that field. Moreover, students can directly contact the experts on a particular topic to consult with them regarding their experience or use them as a source of additional unpublished documents.

Before submitting a dissertation/thesis, the electronic search strategy should be repeated. This process will ensure that the most recently published papers will be considered in the LR.

- Selecting documents for inclusion: Generally, the most recent literature will be included in the form of published peer-reviewed papers. Assess books and unpublished material, such as conference abstracts, academic texts and government reports, are also important to assess since the gray literature also offers valuable information. However, since these materials are not peer-reviewed, we recommend that they are carefully added to the LR.

This task is an important exercise in time management. First, students should read the title and abstract to understand whether that document suits their purposes, addresses the research question, and helps develop the topic of interest. Then, they should scan the full text, determine how it is structured, group it with similar documents, and verify whether other arguments might be considered (5).

### Third step: Analyzing the results

Critical reading and thinking skills are important in this step. This step consists of the following components:

- Reading documents: The student may read various texts in depth according to LR sections and subsections (*defining the main topic*), which is not a passive activity (1). Some questions should be asked to practice critical analysis skills, as listed below. Is the research question evident and articulated with previous knowledge? What are the authors’ research goals and theoretical orientations, and how do they interact? Are the authors’ claims related to other scholars’ research? Do the authors consider different perspectives? Was the research project designed and conducted properly? Are the results and discussion plausible, and are they consistent with the research objectives and methodology? What are the strengths and limitations of this work? How do the authors support their findings? How does this work contribute to the current research topic? (1,19)
- Taking notes: Students who systematically take notes on each document are more readily able to establish similarities or differences with other documents and to highlight personal observations. This approach reinforces the student’s ideas about the next step and helps develop his/her own academic *voice* (1,13). Voice recognition software (16), mind maps (5), flowcharts, tables, spreadsheets, personal comments on the referenced texts, and note-taking apps are all available tools for managing these observations, and the student him/herself should use the tool that best improves his/her learning. Additionally, when a student is considering submitting an LR to a peer-reviewed journal, notes should be taken on the activities performed in all five steps to ensure that they are able to be replicated.

### Fourth step: Writing

The recognition of when a student is able and ready to write after a sufficient period of reading and thinking is likely a difficult task. Some students can produce a review in a single long work session. However, as discussed above, writing is not a linear process, and students do not need to write LRs according to a specific sequence of sections. Writing an LR is a time-consuming task, and some scholars believe that a period of at least six months is sufficient (6). An LR, and academic writing in general, expresses the writer’s proper thoughts, conclusions about others’ work (6,10,13,16), and decisions about methods to progress in the chosen field of knowledge. Thus, each student is expected to present a different learning and writing trajectory.

In this step, writing methods should be considered; then, editing, citing and correct referencing should complete this stage, at least temporarily. Freewriting techniques may be a good starting point for brainstorming ideas and improving the understanding of the information that has been read (1). Students should consider the following parameters when creating an agenda for writing the LR: two-hour writing blocks (at minimum), with prespecified tasks that are possible to complete in one section; short (minutes) and long breaks (days or weeks) to allow sufficient time for mental rest and reflection; and short- and long-term goals to motivate the writing itself (20). With increasing experience, this scheme can vary widely, and it is not a straightforward rule. Importantly, each discipline has a different way of writing (1), and each department has its own preferred styles for citations and references.

### Fifth step: Reflecting on the writing

In this step, the postgraduate student should ask him/herself the same questions as in the *analyzing the results* step, which can take more time than anticipated. Ambiguities, repeated ideas, and a lack of coherence may not be noted when the student is immersed in the writing task for long periods. The whole effort will likely be a work in progress, and continuous refinements in the written material will occur once the writing process has begun.

## LITERATURE REVIEW CHECKLIST

In contrast to review papers, the LR of a dissertation/thesis should not be a standalone piece or work. Instead, it should present the student as a scholar and should maintain the interest of the audience in how that dissertation/thesis will provide solutions for the current gaps in a particular field.

**Figure 3:**
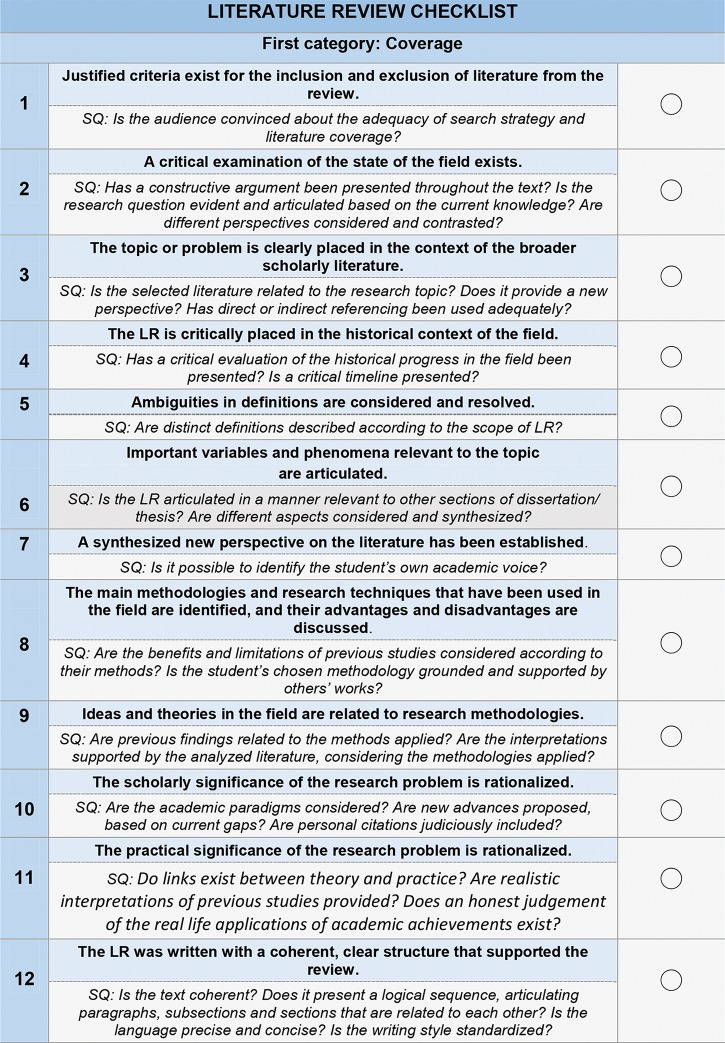
The LR checklist comprises 12 criteria that should ideally be present in the LR section of a dissertation or thesis. Some signaling questions (SQ) are listed below each criterion to facilitate the judgment of whether a particular item was achieved. The checklist represents the learning outcomes of the LR.

A checklist for evaluating an LR is convenient for students’ continuous academic development and research transparency: it clearly states the desired achievements for the LR of a dissertation/thesis. Here, we present an LR checklist developed from an LR scoring rubric (11). For a critical analysis of an LR, we maintain the five categories but offer twelve criteria that are not scaled (Figure 3). The criteria all have the same importance and are not mutually exclusive.

### First category: Coverage

#### 1. Justified criteria exist for the inclusion and exclusion of literature in the review

This criterion builds on the main topic and areas covered by the LR (18). While experts may be confident in retrieving and selecting literature, postgraduate students must convince their audience about the adequacy of their search strategy and their reasons for intentionally selecting what material to cover (11). References from different fields of knowledge provide distinct perspective, but narrowing the scope of coverage may be important in areas with a large body of existing knowledge.

### Second category: Synthesis

#### 2. A critical examination of the state of the field exists

A critical examination is an assessment of distinct aspects in the field (1) along with a constructive argument. It is not a negative critique but an expression of the student’s understanding of how other scholars have added to the topic (1), and the student should analyze and contextualize contradictory statements. A writer’s personal bias (beliefs or political involvement) have been shown to influence the structure and writing of a document; therefore, the cultural and paradigmatic background guide how the theories are revised and presented (13). However, an honest judgment is important when considering different perspectives.

#### 3. The topic or problem is clearly placed in the context of the broader scholarly literature

The broader scholarly literature should be related to the chosen main topic for the LR (*how to develop the literature review* section). The LR can cover the literature from one or more disciplines, depending on its scope, but it should always offer a new perspective. In addition, students should be careful in citing and referencing previous publications. As a rule, original studies and primary references should generally be included. Systematic and narrative reviews present summarized data, and it may be important to cite them, particularly for issues that should be understood but do not require a detailed description. Similarly, quotations highlight the exact statement from another publication. However, excessive referencing may disclose lower levels of analysis and synthesis by the student.

#### 4. The LR is critically placed in the historical context of the field

Situating the LR in its historical context shows the level of comfort of the student in addressing a particular topic. Instead of only presenting statements and theories in a temporal approach, which occasionally follows a linear timeline, the LR should authentically characterize the student’s academic work in the state-of-art techniques in their particular field of knowledge. Thus, the LR should reinforce why the dissertation/thesis represents original work in the chosen research field.

#### 5. Ambiguities in definitions are considered and resolved

Distinct theories on the same topic may exist in different disciplines, and one discipline may consider multiple concepts to explain one topic. These misunderstandings should be addressed and contemplated. The LR should not synthesize all theories or concepts at the same time. Although this approach might demonstrate in-depth reading on a particular topic, it can reveal a student’s inability to comprehend and synthesize his/her research problem.

#### 6. Important variables and phenomena relevant to the topic are articulated

The LR is a unique opportunity to articulate ideas and arguments and to purpose new relationships between them (10,11). More importantly, a sound LR will outline to the audience how these important variables and phenomena will be addressed in the current academic work. Indeed, the LR should build a bidirectional link with the remaining sections and ground the connections between all of the sections (Figure 1).

#### 7. A synthesized new perspective on the literature has been established

The LR is a ‘creative inquiry’ (13) in which the student elaborates his/her own discourse, builds on previous knowledge in the field, and describes his/her own perspective while interpreting others’ work (13,17). Thus, students should articulate the current knowledge, not accept the results at face value (11,13,17), and improve their own cognitive abilities (12).

### Third category: Methodology

#### 8. The main methodologies and research techniques that have been used in the field are identified and their advantages and disadvantages are discussed

The LR is expected to distinguish the research that has been completed from investigations that remain to be performed, address the benefits and limitations of the main methods applied to date, and consider the strategies for addressing the expected limitations described above. While placing his/her research within the methodological context of a particular topic, the LR will justify the methodology of the study and substantiate the student’s interpretations.

#### 9. Ideas and theories in the field are related to research methodologies

The audience expects the writer to analyze and synthesize methodological approaches in the field. The findings should be explained according to the strengths and limitations of previous research methods, and students must avoid interpretations that are not supported by the analyzed literature. This criterion translates to the student’s comprehension of the applicability and types of answers provided by different research methodologies, even those using a quantitative or qualitative research approach.

### Fourth category: Significance

#### 10. The scholarly significance of the research problem is rationalized

The LR is an introductory section of a dissertation/thesis and will present the postgraduate student as a scholar in a particular field (11). Therefore, the LR should discuss how the research problem is currently addressed in the discipline being investigated or in different disciplines, depending on the scope of the LR. The LR explains the academic paradigms in the topic of interest (13) and methods to advance the field from these starting points. However, an excess number of personal citations—whether referencing the student’s research or studies by his/her research team—may reflect a narrow literature search and a lack of comprehensive synthesis of ideas and arguments. 

#### 11. The practical significance of the research problem is rationalized

The practical significance indicates a student’s comprehensive understanding of research terminology (e.g., risk *versus* associated factor), methodology (e.g., efficacy *versus* effectiveness) and plausible interpretations in the context of the field. Notably, the academic argument about a topic may not always reflect the debate in real life terms. For example, using a quantitative approach in epidemiology, statistically significant differences between groups do not explain all of the factors involved in a particular problem (21). Therefore, excessive faith in *p*-values may reflect lower levels of critical evaluation of the context and implications of a research problem by the student.

### Fifth category: Rhetoric

#### 12. The LR was written with a coherent, clear structure that supported the review

This category strictly relates to the language domain: the text should be coherent and presented in a logical sequence, regardless of which organizational (18) approach is chosen. The beginning of each section/subsection should state what themes will be addressed, paragraphs should be carefully linked to each other (10), and the first sentence of each paragraph should generally summarize the content. Additionally, the student’s statements are clear, sound, and linked to other scholars’ works, and precise and concise language that follows standardized writing conventions (e.g., in terms of active/passive voice and verb tenses) is used. Attention to grammar, such as orthography and punctuation, indicates prudence and supports a robust dissertation/thesis. Ultimately, all of these strategies provide fluency and consistency for the text.

Although the scoring rubric was initially proposed for postgraduate programs in education research, we are convinced that this checklist is a valuable tool for all academic areas. It enables the monitoring of students’ learning curves and a concentrated effort on any criteria that are not yet achieved. For institutions, the checklist is a guide to support supervisors’ feedback, improve students’ writing skills, and highlight the learning goals of each program. These criteria do not form a linear sequence, but ideally, all twelve achievements should be perceived in the LR.

## CONCLUSIONS

A single correct method to classify, evaluate and guide the elaboration of an LR has not been established. In this essay, we have suggested directions for planning, structuring and critically evaluating an LR. The planning of the scope of an LR and approaches to complete it is a valuable effort, and the five steps represent a rational starting point. An institutional environment devoted to active learning will support students in continuously reflecting on LRs, which will form a dialogue between the writer and the current literature in a particular field (13).

The completion of an LR is a challenging and necessary process for understanding one’s own field of expertise. Knowledge is always transitory, but our responsibility as scholars is to provide a critical contribution to our field, allowing others to think through our work. Good researchers are grounded in sophisticated LRs, which reveal a writer’s training and long-lasting academic skills. We recommend using the LR checklist as a tool for strengthening the skills necessary for critical academic writing.

## AUTHOR CONTRIBUTIONS

Leite DFB has initially conceived the idea and has written the first draft of this review. Padilha MAS and Cecatti JG have supervised data interpretation and critically reviewed the manuscript. All authors have read the draft and agreed with this submission. Authors are responsible for all aspects of this academic piece.

## Footnotes

1 The questions posed in systematic reviews usually follow the ‘PICOS’ acronym: Population, Intervention, Comparison, Outcomes, Study design.

2 In 1988, Cooper proposed a taxonomy that aims to facilitate students’ and institutions’ understanding of literature reviews. Six characteristics with specific categories are briefly described: Focus: research outcomes, research methodologies, theories, or practices and applications; Goals: integration (generalization, conflict resolution, and linguistic bridge-building), criticism, or identification of central issues; Perspective: neutral representation or espousal of a position; Coverage: exhaustive, exhaustive with selective citations, representative, central or pivotal; Organization: historical, conceptual, or methodological; and Audience: specialized scholars, general scholars, practitioners or policymakers, or the general public.
